# Predictors of inactive disease and remission in children and young adults with juvenile idiopathic arthritis treated with etanercept

**DOI:** 10.1093/rheumatology/keag198

**Published:** 2026-04-20

**Authors:** Vyacheslav Chasnyk, Tamas Constantin, Irina Nikishina, Brigitte Bader-Meunier, Luciana Breda, Pavla Doležalová, Ingrida Rumba-Rozenfelde, Nico Wulffraat, Jonathan Akikusa, Tadej Avcin, Ruben Burgos-Vargas, Jeffrey Chaitow, Luca Carlini, Vassilis Tsekouras, Daniela Graham, Cecilia Borlenghi, Bonnie Vlahos, Chuanbo Zang, Nicolino Ruperto

**Affiliations:** Rheumatology, Saint-Petersburg State Pediatric Medical University, Russian Federation; Unit of Paediatric Rheumatology, Paediatric Centre, Semmelweis University, Budapest, Hungary; Pediatric Department, V.A. Nasonova Research Institute of Rheumatology, Moscow, Russian Federation; Institut IMAGINE, Hôpital Necker-Enfants Malades, Paris, France; Dipartimento di Pediatria, Università degli Studi di Chieti, Chieti, Italy; General University Hospital, Prague and 1st Faculty of Medicine, Charles University, Prague, Czech Republic; Department of Family, Maternal and Child Health, University of Latvia, Riga, Latvia; Department of Pediatric Immunology and Rheumatology, Wilhelmina Children’s Hospital, Utrecht, The Netherlands; Paediatric Rheumatology, Royal Children’s Hospital, Melbourne, Victoria, Australia; University Children’s Hospital, University Medical Center Ljubljana, Ljubljana, Slovenia; Department of Rheumatology, Hospital General de Mexico, Mexico City, Mexico; Paediatric Rheumatology, Sydney Adventist Hospital, Wahroonga, NSW, Australia; Biostatistician Consultant, Utrecht, the Netherlands; Pfizer International Medical Affairs, Cyprus; Pfizer International Medical Affairs, Cyprus; Pfizer International Medical Affairs, Cyprus; Pfizer International Medical Affairs, Cyprus; Pfizer International Medical Affairs, Cyprus; Dipartimento di Medicina e Chirurgia, Università Milano Bicocca, Monza, Italy; Fondazione IRCCS San Gerardo dei Tintori, Reumatologia Pediatrica, PRINTO, Monza, Italy

**Keywords:** juvenile idiopathic arthritis, biologic therapies, adolescent rheumatology, paediatric/juvenile rheumatology, immunosuppressants

## Abstract

**Objectives:**

To identify predictors of clinically inactive disease (CID) and clinical remission (CR) in patients with juvenile idiopathic arthritis receiving etanercept during the 2-year, phase 3b, open-label CLIPPER study (NCT00962741) and the 8-year extension study, CLIPPER2 (NCT01421069).

**Methods:**

Patients with extended oligoarthritis (2–17 years), enthesitis-related arthritis or psoriatic arthritis (each 12–17 years) were enrolled in CLIPPER/CLIPPER2. Predictors of CID (according to Juvenile Arthritis Disease Activity Score [JADAS] and JIA-ACR response criteria) and CR (≥6 months of CID) were identified using a multivariate stepwise logistic regression model.

**Results:**

Two-thirds of patients met the criteria for CID at any point and 34–43% achieved CR. Height *Z*-score ≥0.74, age at onset ≤12 years, normal CRP levels, HLA-B27^+^ status, JADAS low disease activity (LDA) at 3 months and ≤4 swollen joints were predictive of JADAS CID. BMI *Z*-score >0.80, age at onset ≤12 years, normal CRP levels and JADAS LDA at 3 months were predictors of JIA-ACR CID. JADAS LDA at 3 months was a predictor of JADAS CR, and height *Z*-score >1.23, JADAS LDA at 3 months and >12 swollen joints were identified as predictors of JIA-ACR CR.

**Conclusion:**

In patients with JIA treated with etanercept, early responses to treatment in line with treat-to-target recommendations, younger age, HLA-B27^+^ status and lower disease activity at baseline were associated with clinically inactive disease and clinical remission.

**Trial registration:**

ClinicalTrials.gov IDs: CLIPPER (NCT00962741); CLIPPER2 (NCT01421069)

Rheumatology key messages

- In the CLIPPER/CLIPPER2 clinical studies, patients with JIA received etanercept for up to 10 years.
- Predictors of clinically inactive disease or clinical remission were identified in stepwise logistic regression analyses.
- Predictors identified were early responses to treatment, younger age, HLA-B27^+^ status and lower disease activity.

## Introduction

Juvenile idiopathic arthritis (JIA) is a general term for a heterogeneous group of chronic childhood arthritic diseases that are grouped into mutually exclusive categories: systemic, oligoarticular (extended or persistent), polyarticular RF-negative or RF-positive, psoriatic arthritis (PsA), enthesitis-related arthritis (ERA) and undifferentiated JIA [1–3]. Treatment options include disease modifying anti-rheumatic drugs (DMARDs) such as conventional synthetic DMARDs (csDMARDs), targeted synthetic DMARDs (tsDMARDs) and biologic DMARDs (bDMARDs). The efficacy and safety of bDMARDs and tsDMARDs in patients with JIA have been demonstrated in numerous phase 3 trials for TNF inhibitors etanercept [4–8], adalimumab [8, 9], infliximab [10] and golimumab [11], anti-IL-1β mAb canakinumab [12], anti-IL-6 receptor mAb tocilizumab [13–15], anti-CD80/CD86 mAb abatacept [16] and Janus kinase inhibitors tofacitinib [17] and baricitinib [18]. Etanercept (ETN) has been studied for the treatment of different categories of JIA namely polyarthritis and extended oligoarthritis (eoJIA), and enthesitis-related arthritis (ERA) [19]. In the 2-year, phase 3b, open-label CLIPPER study (NCT00962741) and 8-year CLIPPER 2 extension study (NCT01421069), treatment with ETN was generally well tolerated and led to durable responses in patients with eoJIA, ERA or PsA [4–7]. Understanding baseline predictors of clinically inactive disease (CID) and clinical remission (CR; CID ≥6 months) would enable a more individualized approach to the treatment of JIA. The aim of this post-hoc analysis was to identify baseline predictors of CID and CR at any time point during the CLIPPER studies.

## Methods

### Study design and population

The study design and inclusion criteria are described in detail elsewhere [4–6]. Briefly, CLIPPER enrolled patients with eoJIA (aged 2–17 years), ERA or PsA (each aged 12–17 years). Patients who received ≥1 dose of ETN (0.8 mg/kg QW [max 50 mg/week]) and completed CLIPPER could enter CLIPPER2 and receive ETN (0.8 mg/kg QW [max 50 mg/week]) for ≤10 years across both studies.

Participants who achieved JIA-ACR CID for ≥6 continuous months on ETN or who, in the investigator’s judgment, had a good clinical response or were likely to benefit from withdrawal could enter (per investigator and study participant decision) the optional withdrawal/re-treatment period. Participants who entered the withdrawal period were offered the option to restart ETN if required (per investigator’s judgment). Patients who did not complete 24 months of active treatment in CLIPPER or discontinued ETN for any reason before the end of CLIPPER2, as well as those in the withdrawal period who were ineligible for retreatment with ETN or who discontinued during the retreatment period, were eligible to enrol into the observational period of CLIPPER2 and were assessed for safety only every 6 months until the end of the trial. Participants in the observational period could not resume ETN treatment during the study but could receive standard of care, including any anti-TNF agents (e.g. commercial ETN) and/or other immunosuppressive biologic agents at the discretion of the investigator.

Participating study centres were part of the Paediatric Rheumatology International Trials Organisation (PRINTO) [20] (Supplementary Table S1). The CLIPPER/CLIPPER 2 studies were approved by the relevant regulatory bodies of each country and institution and were conducted in compliance with the Declaration of Helsinki, all International Council for Harmonisation Good Clinical Practice Guidelines, and local regulatory requirements. The independent ethics committees or institutional review boards involved are listed in the supplementary materials. All participants or their parents/guardians provided written informed consent prior to participating in any study activities.

### Efficacy assessment

Efficacy assessments included the JIA core set of outcomes (physician’s global assessment [PGA] of disease activity, parent’s/patient’s global assessment of overall well-being, functional ability, joint count with active arthritis, joint count with restricted motion, and ESR) [21]. CID was defined according to the Juvenile Arthritis Disease Activity Score (JADAS) and the JIA-ACR response criteria. The 2012–2014 JADAS 10 criteria [22] define CID a score for polyarthritis and oligoarthritis ≤1 based on four components: PGA of disease activity; patient’s/parent’s global assessment; number of joints with active arthritis; and C-reactive protein (CRP). CID according to JIA-ACR (Wallace) criteria [23] is defined as no joints with active arthritis; no fever, rash, serositis, splenomegaly or generalized lymphadenopathy attributable to JIA; no active uveitis; CRP level within normal limits or, if elevated, not attributable to JIA; PGA of disease activity score of best possible; and duration of morning stiffness ≤15 min. CR was defined as ≥6 continuous months of CID on medication according to either set of criteria.

### Statistical analysis of independent predictors of CID and CR

Predictors of CID and CR were identified using a stepwise logistic regression model, with CR defined according to JADAS or JIA-ACR criteria as the dependent variable and demographic and baseline disease characteristics and JIA category as independent variables. The value cutoff for inclusion in the stepwise regression model was set as *P* ≤ 0.05. The analyses included the following pre-defined demographic and baseline disease characteristics and JIA type: sex, *Z*-scores for weight, BMI and height (determined using age- and sex-matched reference data from the World Health Organization) [24], disease duration, HLA-B27, number of active joints, number of joints with limitation of motion (LOM), number of painful joints, number of swollen joints, CRP, Childhood Health Assessment Questionnaire (CHAQ), parent’s/patient’s assessment of overall well-being, race, age at disease onset, morning stiffness, tender entheseal score, overall and nocturnal back pain visual analogue scale (VAS) score, modified Schober’s test (ERA only), psoriasis body surface area (BSA; PsA only), PGA of psoriasis (PsA only), and baseline concomitant therapies (DMARDs, methotrexate, sulfasalazine, chloroquine, hydroxychloroquine, oral corticosteroids, NSAIDs). Pubertal status was not considered. Continuous variables were dichotomized based on receiver operating characteristic curves [25]. Early markers of response were also analysed as independent variables; these included JADAS CID (≤1) at week 4, week 8 and 3 months, JADAS low disease activity (LDA) at 3 months (defined as number of joints with active arthritis ≤4 and JADAS ≤2, or number of joints with active arthritis >4 and JADAS ≤3.8), JIA-ACR CID at week 4, week 8 and 3 months, and CRP (normal *vs* high) at 3 months. Baseline characteristics associated with CID and CR were also identified using univariate logistic analyses. All analyses were done in the modified intent-to-treat population of patients who received at least one dose of ETN. Observed data were used for the analyses. There was no imputation for missing data, and no sensitivity analyses.

## Results

### Patients

Of 127 patients enrolled in CLIPPER (eoJIA: *n *= 60; ERA: *n *= 38; PsA: *n *= 29), [6] 109 (86%) entered CLIPPER2 and 84 (66%) completed the extension study (27 on active treatment and five on re-treatment) (see Horneff *et al*. [6] and Supplementary Fig. S1 for subject disposition**)**. Demographics and baseline characteristics are described in Table 1. Mean age at study start was 12 years, 57% of patients were female and mean disease duration was 27 months [6]. Eighty-six percent of patients were taking a sDMARD at baseline with 68% taking MTX. Mean duration of treatment with ETN was 245 weeks with a mean weekly dose of 42 mg. The total exposure was 683 patient-years. A total of 90/127 (71%) and 82/127 (65%) of patients achieved CID according to JADAS and JIA-ACR criteria (54/127 [43%] and 43/127 [34%] for CR), respectively, at any point during CLIPPER/CLIPPER2 (Table 2).

**Table 1:** Baseline characteristics at the start of CLIPPER.

Parameter	eoJIA	ERA	PsA	Total
	(*n* = 60)	(*n* = 38)	(*n* = 29)	(*n* = 127)
Age at enrolment, years	8.6 (4.6)	14.5 (1.6)	14.5 (2.0)	11.7 (4.5)
Disease duration, months	31.6 (31.7)	23.0 (19.8)	21.8 (20.2)	26.8 (26.4)
Female, *n* (%)	41 (68.3)	8 (21.1)	23 (79.3)	72 (56.7)
Height *Z*-score	0.06 (1.17) (*n* = 58)	0.24 (0.89)	0.41 (1.06)	0.19 (1.07) (*n* = 125)
Weight *Z*-score	0.08 (1.10)	−0.05 (0.74)	0.63 (1.05)	0.17 (1.02)
BMI *Z*-score	0.00 (1.28)	−0.29 (0.87)	0.51 (1.17)	0.03 (1.17)
JIA core set				
PGA of disease activity VAS	5.0 (1.8)	5.4 (1.9)	4.7 (1.4)	5.0 (1.8)
Number of active joints	7.6 (5.1)	5.2 (3.6)	7.0 (4.3)	6.7 (4.6)
Number of joints with LOM	6.3 (4.4)	4.8 (4.0)	5.6 (4.1)	5.7 (4.2)
CRP, mg/l	6.3 (10.6)	15.3 (21.5)	3.2 (4.7)	8.2 (14.7)
PtGA score	4.8 (2.4)	5.4 (2.3)	4.6 (2.2)	5.0 (2.3)
CHAQ score	0.9 (0.7)	0.7 (0.5)	0.7 (0.6)	0.8 (0.6)
JADAS 73 score	17.9 (7.5)^a^	17.0 (7.1)^a^	15.9 (5.4)^a^	17.2 (7.0)^a^
Additional measures				
Overall back pain VAS, mm	—	25.9 (28.0)	—	—
Nocturnal back pain VAS, mm	—	16.4 (27.8)	—	—
Psoriasis BSA, %	—	—	10.4 (13.4)	—
PGA of psoriasis	—	—	1.8 (1.4)	—
Baseline therapies				
Any sDMARD	54 (90.0)	32 (84.2)	23 (79.3)	109 (85.8)
Methotrexate	49 (81.7)	18 (47.4)	19 (65.5)	86 (67.7)
Oral glucocorticoids^b^	7 (11.7)	8 (21.1)	1 (3.5)	16 (12.6)
Oral NSAID	32 (53.3)	26 (68.4)	16 (55.2)	74 (58.3)

a Values are reported as mean (s.d.) unless otherwise indicated. ‘Efficacy and safety of open-label etanercept on extended oligoarticular juvenile idiopathic arthritis, enthesitis-related arthritis and psoriatic arthritis: part 1 (week 12) of the CLIPPER study’ is adapted from Horneff *et al* [6]. , licensed under CC BY-NC 3.0. *n *= 55 (eoJIA), 38 (ERA), 26 (PsA) and 119 (total).

b ≤0.2 mg/kg/day or 10 mg/day (whichever was less). BSA: body surface area; CHAQ: Childhood Health Assessment Questionnaire; eoJIA: extended oligoarticular juvenile idiopathic arthritis; ERA: enthesitis-related arthritis; JADAS: Juvenile Arthritis Disease Activity Score; LOM: limitation of motion; PGA: physician’s global assessment; PtGA: parent’s global assessment; sDMARD: synthetic DMARD; VAS: visual analogue scale.

**Table 2:** Proportions of patients with CID or CR (first time achieved) during CLIPPER/CLIPPER2.

	JIA-ACR	JADAS 10
	(*n* = 127)	(*n* = 127)
CID	82 (64.57)	90 (70.87)
CR on medication: ≥6 continuous months of CID on DMARDs	43 (33.86)	54 (42.52)
CR off medication: ≥12 continuous months of CID off DMARDs	4 (3.15)	3 (2.36)
CR on medication: ≥2 years of continuous CID on DMARDs	13 (10.24)	26 (20.47)
CR on medication: ≥5 years of continuous CID on DMARDs	2 (1.57)	6 (4.72)
CR on medication: ≥6 continuous months of CID on DMARDs at year 2	23 (18.11)	43 (33.86)
CR on medication: ≥6 continuous months of CID on DMARDs at year 5	28 (22.05)	53 (41.73)

Values are reported as *n* (%). CID: clinically inactive disease; CR: clinical remission; JADAS: Juvenile Arthritis Disease Activity Score.

#### Predictors of CID and CR

**Figure 1:**
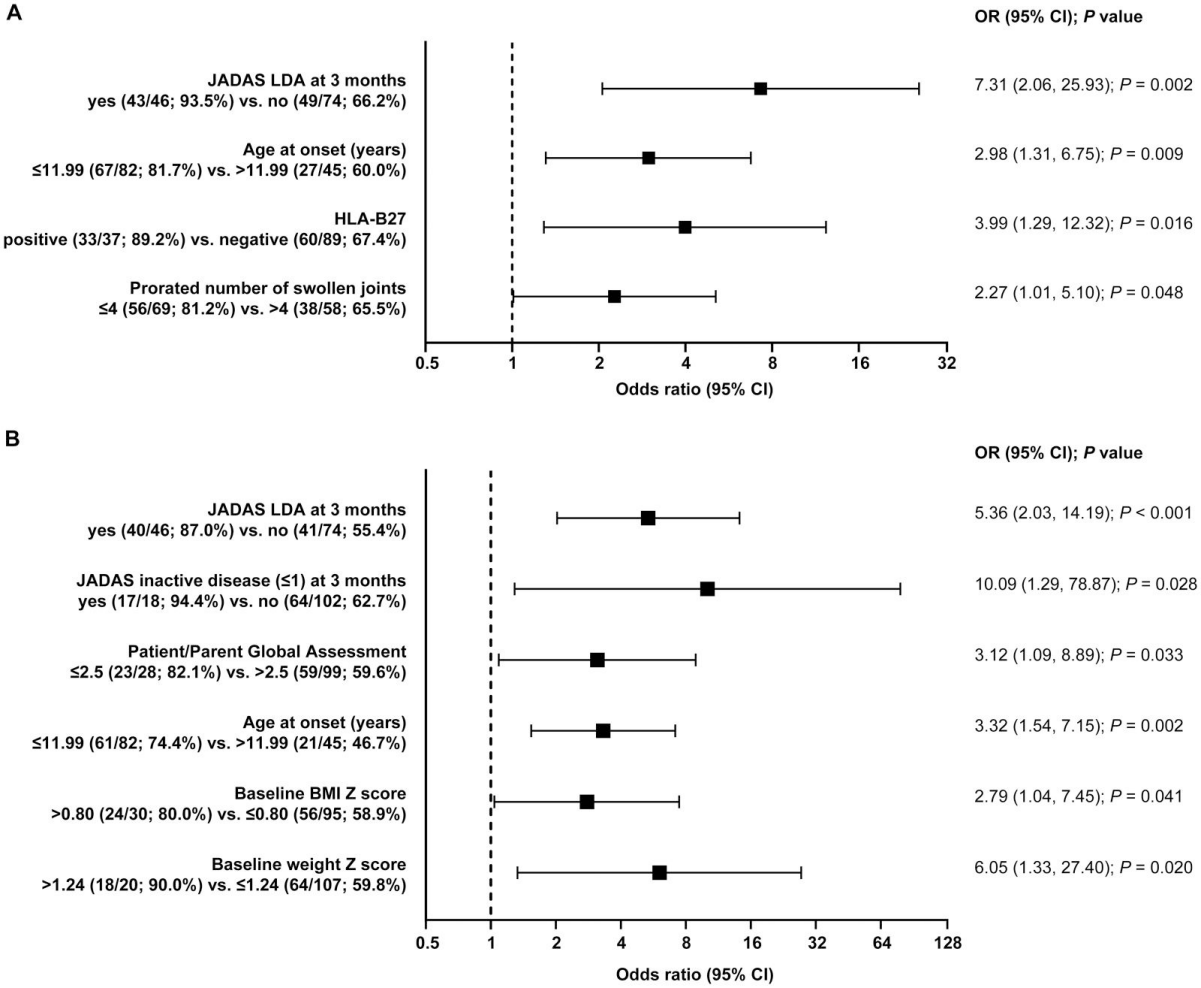
Baseline and 3 month factors associated with CID according to (**A**) JADAS and (**B**) JIA-ACR criteria according to the univariate analysis. Significant results (*P* ≤ 0.05) from the univariate analyses are shown. CID: clinically inactive disease; JADAS: Juvenile Arthritis Disease Activity Score; LDA: low disease activity; OR: odds ratio

**Figure 2:**
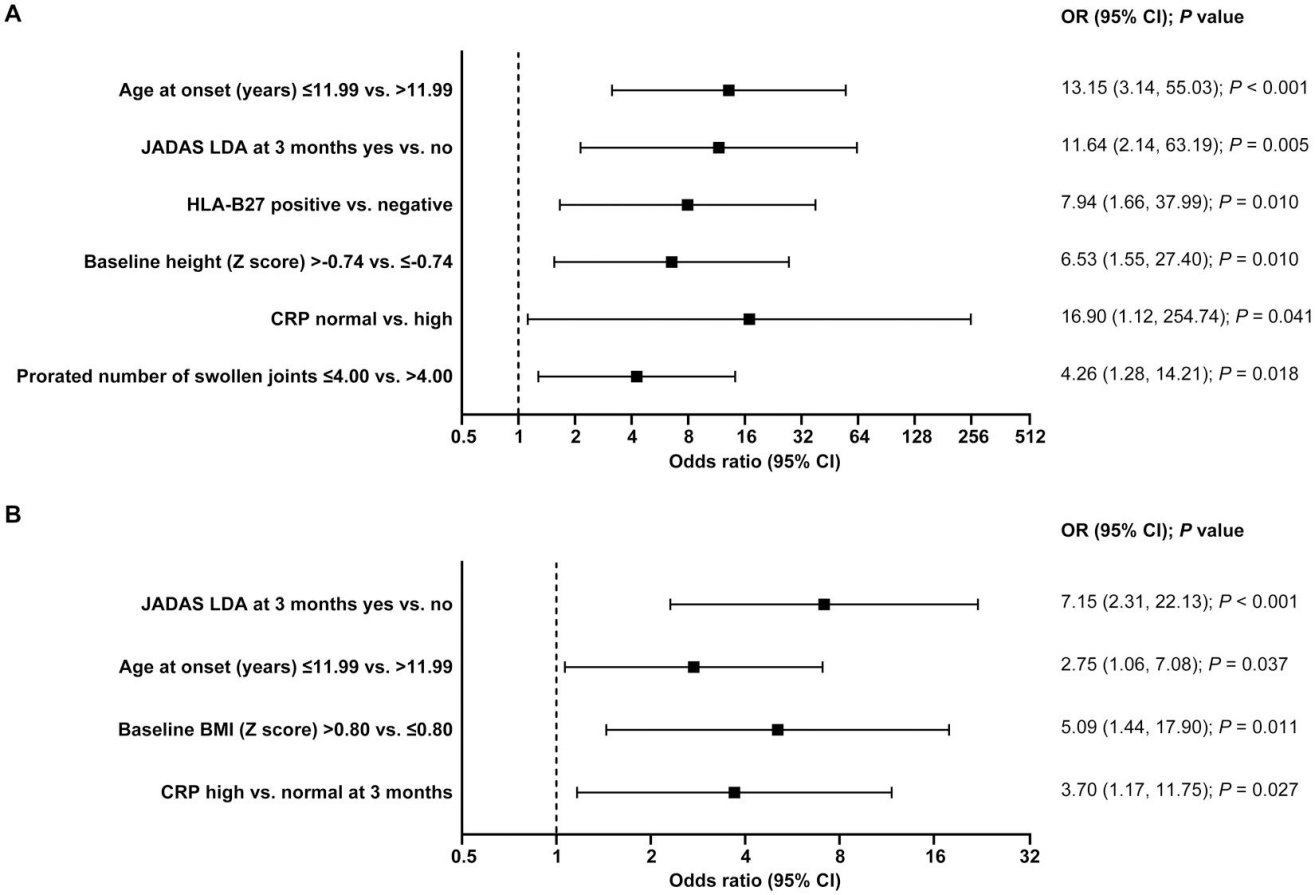
Predictors of CID according to (**A**) JADAS and (**B**) JIA-ACR criteria, according to the multivariate analysis. Data are based on final results of the stepwise model. *P*-value to enter: 0.15. *P-*value to stay: 0.05. CID: clinically inactive disease; JADAS: Juvenile Arthritis Disease Activity Score; LDA: low disease activity; OR: odds ratio

**Figure 3:**
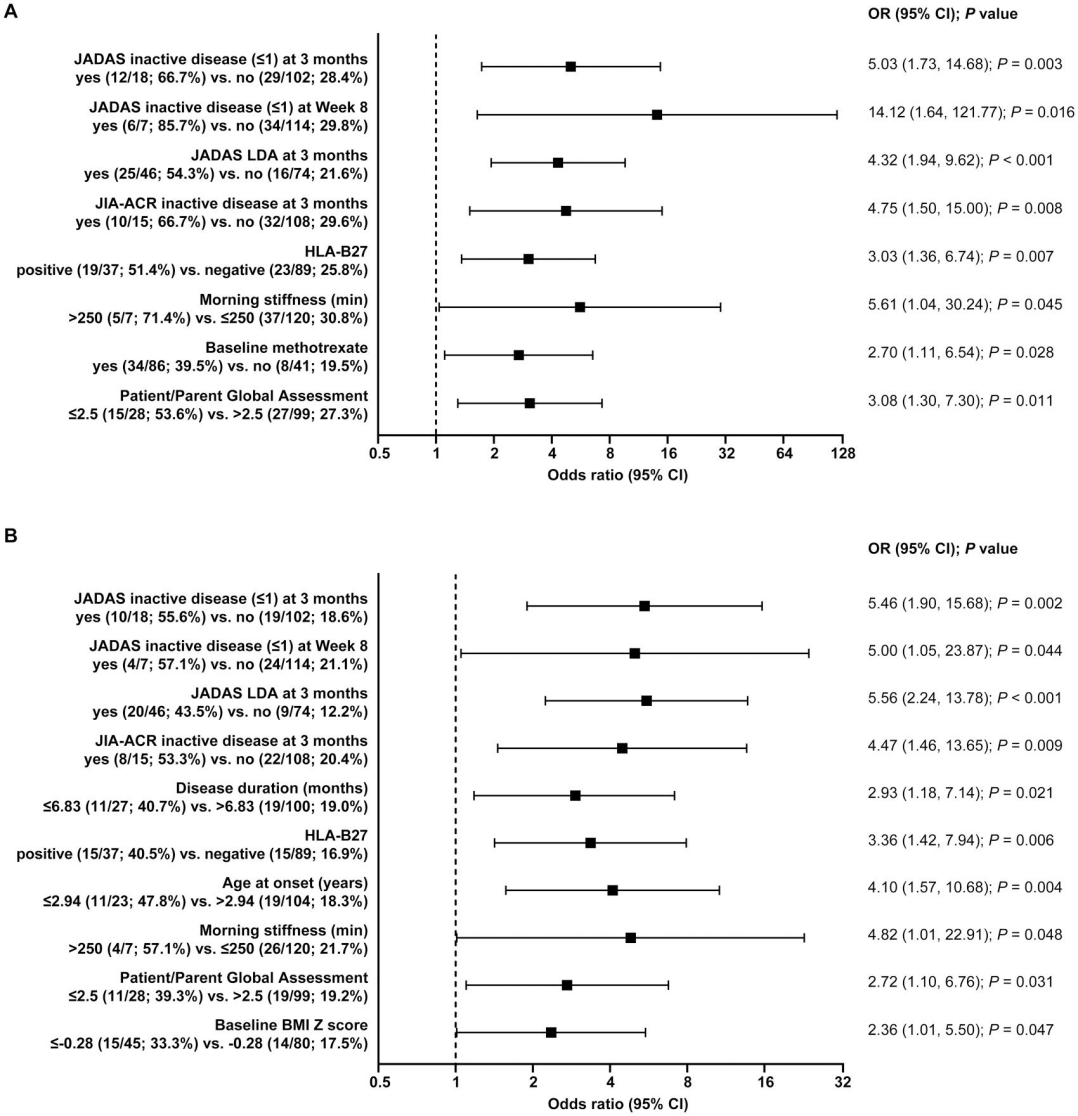
Baseline factors associated with CR according to (**A**) JADAS and (**B**) JIA-ACR criteria according to the univariate analysis. Significant results (*P* ≤ 0.05) from the univariate analyses are shown. CR: clinical remission; JADAS: Juvenile Arthritis Disease Activity Score; LDA: low disease activity; OR: odds ratio

**Figure 4:**
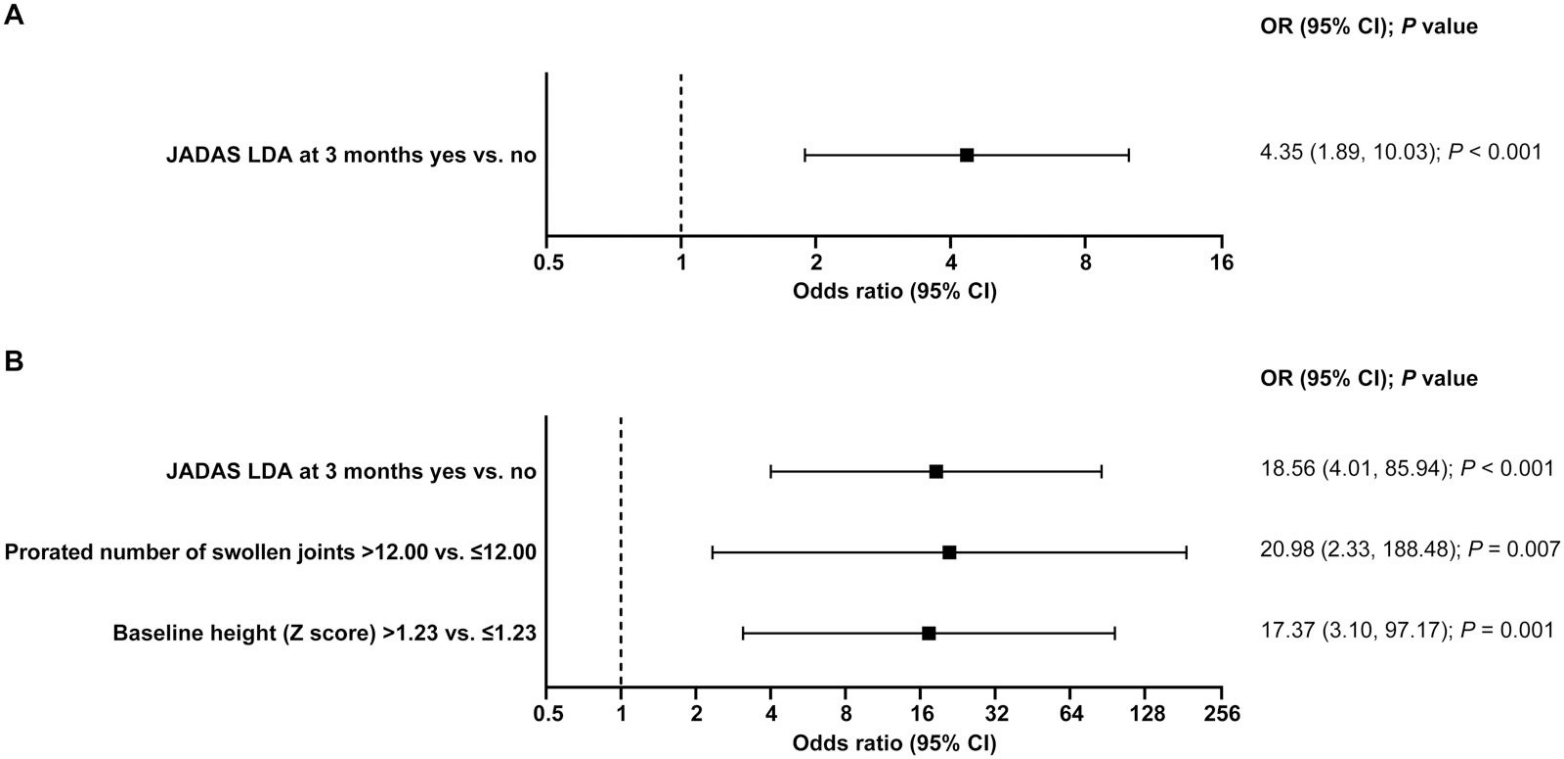
Predictors of CR according to (**A**) JADAS and (**B**) JIA-ACR criteria as per the stepwise regression according to the multivariate analysis. *P*-value to enter: 0.15. *P*-value to stay: 0.05. CR: clinical remission; JADAS: Juvenile Arthritis Disease Activity Score; LDA: low disease activity; OR: odds ratio

Factors significantly associated with CID and CR in univariate analyses are presented in Figs 1 and 3 and Supplementary Tables S2 and S3, available at *Rheumatology* online, and response predictors identified in the stepwise multivariate analyses are presented in Figs 2 and 4 and Supplementary Tables S4 and S5, available at *Rheumatology* online.

### Predictors of CID according to JADAS and JIA-ACR criteria

In the univariate analyses, the following characteristics were associated with CID at any point during the study according to JADAS criteria (odds ratio [OR] [95% CI]): JADAS LDA at 3 months (7.31 [2.06, 25.93], *P* = 0.002); age at onset ≤11.99 years (2.98 [1.31, 6.75], *P* = 0.009), as well as HLA-B27 positivity and ≤4 swollen joints (prorated) at baseline (Fig. 1A).

Similarly, JADAS LDA (5.36 [2.03, 14.19], *P* < 0.001) and CID at 3 months (10.09 [1.29, 78.87], *P* = 0.028), plus patient/parent global assessment score ≤2.5, age at onset ≤12, and baseline BMI and weight *Z*-scores were associated with CID according to JIA-ACR criteria (Fig. 1B).

In the stepwise multivariate regression model, predictors for JADAS CID were age at onset ≤11.99 years (13.15 [3.14, 55.03], *P* < 0.001), JADAS LDA at 3 months (11.64 [2.14, 63.19], *P* = 0.005), HLA-B27 positive, height *Z*-score, normal CRP levels, and ≤4 swollen joints (Fig. 2A).

Multivariate predictors according to JIA-ACR criteria were JADAS LDA at 3 months (7.15 [2.31, 22.13], *P* < 0.001), age at onset ≤12 years (2.75 [1.06, 7.08], *P* = 0.037), followed by BMI *Z*-score and high CRP levels at baseline (Fig. 2B). All 127 patients were included in the models. Non-significant characteristics were removed by stepwise regression and only significant characteristics remained in the final model.

### Predictors of CR according to JADAS and JIA-ACR criteria

In the univariate analyses, the following characteristics were associated with achieving CR according to JADAS criteria (Fig. 3A, Supplementary Table S3): JADAS CID at 3 months (5.03 [1.73, 14.68], *P* = 0.003), at week 8 (14.12 [1.64, 121.77], *P* = 0.016), JADAS LDA at 3 months (4.32 [1.94, 9.62], *P* < 0.001), and JIA-ACR CID at 3 months (4.75 [1.50, 15.00], *P* = 0.008), HLA-B27 positivity, morning stiffness >250 min at baseline, baseline methotrexate use and patient/parent global assessment score ≤2.5 (Fig. 3A).

Characteristics associated with CR according to JIA-ACR criteria were similar to the JADAS CR predictors. In particular the strongest ones were JADAS CID at 3 months (5.46 [1.90, 15.68], *P* = 0.002), at week 8 (5.00 [1.05, 23.87], *P* = 0.044), LDA at 3 months (5.56 [2.24, 13.78], *P* < 0.001), and JIA-ACR CID at 3 months (4.47 [1.46, 13.65], *P* = 0.009) followed by disease duration, HLA-B27 positivity, age at onset ≤3 years, baseline morning stiffness >250 min, baseline patient/parent global assessment score ≤2.5, and baseline BMI *Z*-score ≤−0.28 (Fig. 3B).

In the stepwise regression model, the following predictors for CR (OR [95% CI]) were identified (Fig. 4A): JADAS LDA at 3 months (4.35 [1.89, 10.03], *P* < 0.001) for CR according to JADAS criteria and JADAS LDA at 3 months (18.56 [4.01, 85.94], *P* < 0.001), >12 swollen joints at baseline (prorated; 20.98 [2.33, 188.48], *P* = 0.007), and baseline height (*Z*-score) >1.23 (17.37 [3.10, 97.17], *P* = 0.01) for CR according to JIA-ACR criteria (Fig. 4B).

## Discussion

Patients in the CLIPPER studies received ETN for up to 10 years, providing long-term data collected within a clinical trial setting. This is especially important in JIA, since patients will take treatments for several decades. In this post-hoc analysis, several characteristics associated with or predictive of CID or CR were identified.

Early responses to treatment were associated with and predictive of CR: JADAS CID at week 8 and CID or at least LDA at 3 months were associated with CR according to JADAS and JIA-ACR criteria. JADAS CID at 3 months was predictive for CR according to either set of criteria. In addition, JADAS LDA at 3 months was both associated with and predictive of CID (JADAS and JIA-ACR) at any point during the study. Benefits of achieving LDA early during treatment include improved quality of life and reduced risk of long-term joint damage [26, 27], and rapid response to treatment is a feature of ‘easy-to-control’ disease [28]. Generally, responses to treatment (CID or LDA) as measured every 3 months after treatment initiation are used to inform future treatment strategies to ensure optimal outcomes for patients (treat-to-target approach) [29–31]. Observational data from the BiKeR registry showed that response to ETN during the first 6 months of treatment was associated with a higher likelihood of discontinuation of treatment following CID [32]. These observations are in line with the treat-to-target recommendation in JIA, which suggests abatement of disease activity in the earlier phase of treatment [29].

Age at disease onset of <12 years was both associated with and identified as a predictor of CID (using either set of criteria), and age at onset of <3 years was associated with JIA-ACR CR. An Italian prospective study also reported that younger age at onset (<3.6 years) was associated with JIA-ACR CID [33].

In this post-hoc analysis, CID and CR were more likely in patients who were HLA-B27 positive. The HLA-B27 antigen is associated with higher prevalence of rheumatic and other autoimmune diseases in adults as well as younger patients [34, 35] and more severe disease [34, 36]. In a retrospective analysis from Poland with up to 18 years of follow-up, HLA-B27^+^ patients with JIA were more likely to receive bDMARDs [36], and in a Latvian observational study, the specific HLA-B27 allele determined response to ETN treatment [37]. Data from the BiKeR registry reported HLA-B27^+^ status was associated with JIA-ACR90 response [38]. HLA-B27 as a predictor for the attainment of CID and CR likely reflects the clinical observation that HLA-B27 positive ERA patients have a more undifferentiated arthritis with peripheral involvement that more easily responds to therapy.

Generally, lower disease activity (fewer swollen joints, lower patient’s/parent’s global assessment scores and normal CRP levels) at baseline was associated with and predictive of both CID and CR. This observation is in line with observations in JIA using these or other measures of disease activity [33, 38–40]. Notable exceptions in our study were a longer duration of morning stiffness, which was associated with CR, and swollen joint count of >12, which was predictive of JIA-ACR CR, both indicating greater disease severity.

Results for height, weight and BMI (a function of height and weight) at baseline were contradictive with lower weight and BMI being associated with JIA-ACR CID, while higher height and BMI were identified as predictors for JADAS CID, and higher height was predictive of JIA-ACR CR. Few studies identified any of these factors as predictors of response [41] and, as these factors can vary widely, particularly so in children, they are unlikely to be reliable predictors of response. Height as a predictor for inactive disease and clinical remission likely acts as an indirect marker of shorter disease duration and wider and earlier use of biologic agents, especially in higher-resourced countries. It is indeed known that better control of disease activity is coupled with increased height [42].

JADAS CR was more likely in patients receiving MTX at baseline. Some studies in JIA have reported the impact of MTX use on response to ETN treatment. In a German study, concomitant MTX improved the effectiveness of TNF inhibitors in patients with polyarticular JIA [43]. One Canadian study of patients receiving MTX that compared data from four clinical trials reported that fewer joints were affected at bDMARD treatment initiation; patients with fewer affected joints were more likely to respond to bDMARDs, although no direct relationship between MTX use and response was identified [44].

Study limitations included the post-hoc nature of this analysis and the lack of a control group (patients on other biologics or standard therapy), thus limiting the ability to draw conclusions about the specificity of the outcomes relating to ETN treatment, or whether they reflect the underlying disease course. A similar analysis with other advanced therapies would help to address this limitation of the study. These analyses focused on CID and CR at any time during the study, and no conclusions on long-term CR can be drawn. Due to small sample sizes, we also did not stratify the data by JIA subtypes, which have differing disease biology. Hence, some caution may be needed when interpreting and generalizing the findings to specific disease subtypes. Any similar future studies of advanced treatments for JIA could also stratify by pubertal age to enhance clinical relevance. JADAS criteria [22, 45] for CID have been updated since the inception of the CLIPPER study, and were not included in the analysis; however, we do not expect that this will bear any significant impact on our findings considering the minor differences between the older and newer criteria. Strengths of our study include the time frame of up to 10 years of follow-up and data that were derived from a clinical study. A useful clinical application of the identified predictors will guide, in an evidence-based manner, early treatment intensification, tapering or change therapy strategies, as well as monitoring intervals as per the treat to target JIA recommendations [29].

## Conclusions

In patients with JIA treated with etanercept, early responses to treatment in line with the treat-to-target recommendation, younger age at onset, HLA-B27 positivity and lower disease activity at 3 months were associated with CID and CR.

## Supplementary Material

keag198_Supplementary_Data

## Data Availability

Upon request, and subject to review, Pfizer will provide the data that support the findings of this study. Subject to certain criteria, conditions and exceptions, Pfizer may also provide access to the related individual de-identified participant data. See https://www.pfizer.com/science/clinical-trials/trial-data-and-results for more information.
