# Supplementary material for: Predictors of inactive disease and remission in children and young adults with juvenile idiopathic arthritis treated with etanercept
Source: Rheumatology (Oxford). 2026 Apr 20;65(5):keag198. doi: 10.1093/rheumatology/keag198 (PMC13220752; doi:10.1093/rheumatology/keag198)
Supplement: keag198_Supplementary_Data [file keag198_supplementary_data.pdf]

Supplementary Materials for

Predictors of inactive disease and remission in children and young adults with juvenile idiopathic arthritis treated with etanercept

Vyacheslav Chasnyk, Tamas Constantin, Irina Nikishina, Brigitte Bader-Meunier, Luciana Breda, Pavla Dolezalova, Ingrida Rumba-Rozenfelde, Nico Wulffraat, Jonathan Akikusa, Tadej Avcin, Ruben Burgos-Vargas, Jeffrey Chaitow, Luca Carlini, Vassilis Tsekouras, Daniela Graham, Cecilia Borlenghi, Bonnie Vlahos, Chuanbo Zang, Nicolino Ruperto for the Paediatric Rheumatology International Trials Organisation (PRINTO)

Contents

Independent ethics committees or institutional review boards………………………………………………………………………………………………2

Supplementary Table S1. CLIPPER/CLIPPER2 investigators 3

Supplementary Table S2. All baseline variables tested for association with CID – univariate analysis 5

Supplementary Table S3. All baseline variables tested for association with CR – univariate analysis 9

Supplementary Table S4. Significant predictors for CID – multivariate analysis 13

Supplementary Table S5. Significant predictors for CR – multivariate analysis 13

Supplementary Figure S1. Subject disposition CLIPPER 2……………………………………………………………………………………………14

Independent ethics committees or institutional review boards

Royal Children's Hospital, Royal Children's Hospital Ethics in Human Research Committee, Flemington Road, Parkville, VIC 3052; Royal Alexandra Hospital for Children, Human Research Ethics Committee, The Children's Hospital at Westmead, Research and Development Office, Corner Hawkesbury Road and Hainsworth Street, Westmead, NSW 2145; Universitair Ziekenhuis Gent, Ethisch Comite De Pintelaan 185 Gent, 9000; Comite de Etica de la investigacion, Riesgo de Fractura S.A, Carerra 13 N° 97-25, Bogota, Cundinamarca; Comite de ética en Investigación de Servimed E.U Calle 51 No. 34-17, Centro comercial Cabecera Etapa I, Bucaramanga, Santander; Comité de etica independiente centro de reumatologia y ortopedia Cr. 49C No. 82-120, Barranquilla, Atlantico; Eticka komise Revmatologicky ustav Na Slupi 4 Praha 2, 128 50; Eticka komise Vseobecne fakultni nemocnice v Praze Na Bojisti 1 Praha 2, 128 08; Eticka komise FN Brno Jihlavská 20 Brno, 625 00; Comité de protection des personnes <Ile-de-France II> Hopital Necker Enfants malades 45, rue des Saint Pères PARIS, 75006; Ethik-Kommission der Aerztekammer Nordrhein Tersteegenstrasse 9 Duesseldorf, 40474; Egeszsegugyi Tudomanyos Tanacs Klinikai Farmakologiai Etikai Bizottsaga Arany J. u. 6-8. Budapest, 1051; Comitato Etico dell'Universita' degli Studi Gabriele D'Annunzio e della ASL 2 Lanciano-Vasto-Chieti di Chieti Via dei Vestini, 31 Chieti, 66100; Ethics Committee of the LU Scientific Institute of Cardiology for Clinical & Physiological Research and Drug and Pharmaceutical Product Clinical Investigation Cardiology Institute of Latvia Pilsonu Street 13 Riga, 1002; Lithuanian Bioethics Committee Didzioji 22 Vilnius, 01128; Comite Bioetico para la Investigacion Clinica S.C. Puebla 422 Despacho 4 Col. Roma Sur, DISTRITO FEDERAL 06700; Universitair Medisch Centrum Utrecht Medisch Ethische Toetsingscommissie Heidelberglaan 100 Utrecht, 3584 CX; Regional komite for medisinsk og helsefaglig forskningsetikk Sør- Øst C (REK sør-Øst C) Postboks 1130 Blindern Oslo, 0318; Komisja Bioetyczna przy Intytucie Reumatologii im. prof. dr hab. med. Eleonory Reicher ul. Spartanska 1 Warszawa, 02-637; Komisja Etyczna Badan Naukowych Przy Instytucie Reumatologii Ul Spartanska 1 Warszawa, 02 637; Ethics Committee of the Research Institute of Rheumatology of Russian Academy of Medical Science Kashirskoe shosse, 34 Moscow, 115522; Ethics Council at the Ministry of Healthcare and Social Development of Russian Federation 3, Rakhmanovskij per. Moscow, 127994; Ethics Committee at St.Petersburg State Pediatric Medical Academy of Minzdravsotsrazvitiya of Russia 2 ulitsa Litovskaya Saint-Petersburg, 194100; Ethics Committee Clinical Centre of Nis Clinical Center Nis Bulevar dr. Zorana Djindjica 48 Nis, 18000; Ethics Committee Institute for Rheumatology Resavska 69 Belgrade, 11000; Eticka komisia DFN Kosice Detska fakultna nemocnica Kosice Trieda SNP 1 Kosice, 040 01; Eticka komisia, Narodny ustav reumatickych chorob Nabrezie I. Krasku 4 Piestany, 921 12; The National Medical Ethics Committee of the Republic of Slovenia Zaloska 7 Ljubljana, 1525; Hospital Sant Joan de Deu Comité Etico De Investigatcion Clinica Edificio Docente C/ Santa Rosa, 39-57. 4ª Planta Esplugues De Llobregat, Barcelona 08950; Hospital Sant Joan de Deu Comite Etico de Investigacion Clinica Passeig Sant Joan de Deu, 2 Esplugues de Llobregat, Barcelona 08950; Hospital Universitario La Fe Comite Etico de Investigacion Clinica Av. Campanar, nº 21 Valencia, Valencia 46009; Hospital Ramon y Cajal Comite Etico de Investigacion Clinica Planta -2. Dcha. Ctra. de Colmenar Viejo, Km. 9,1 Madrid, Madrid 28034.

Supplementary Table S1. CLIPPER/CLIPPER2 investigators

Country	Town	Principal Investigator Name
Australia	Melbourne	Jonathan Akikusa
Australia	Sydney	Jeffrey Chaitow
Belgium	Gent	Joke Dehoorne
Belgium	Bruxelles	Bernard Lauwerys
Belgium	Leuven	Carine Wouters
Colombia	Barranquilla	Juan Jose Jaller Raad
Colombia	Bucaramanga	William Jose Otero Escalante
Colombia	Bogota	Patricia Julieta Velez
Czech Republic	Prague	Pavla Dolezalova
Czech Republic	Praha 2	Katerina Jarosova
Czech Republic	Brno	Marie Macku
France	Paris	Brigitte Bader Meunier
France	Paris	Chantal Job-Deslandre
Germany	Hamburg	Ivan Foeldvari
Germany	Sankt Augustin	Gerd Horneff
Germany	Bremen	Hans-Iko Huppertz
Germany	Berlin	Ralf Trauzeddel
Hungary	Budapest	Tamas Constantin
Italy		Luciana Breda
Italy	Genoa	Nicolino Ruperto
Latvia	Riga	Valda Stanevica
Latvia	Riga	Ingrida Rumba-Rozenfelde
Lithuania	Vilnius	Violeta Panaviene
Mexico	Mexico City	Ruben Burgos-Vargas
Netherlands	Utrecht	Nico Wulffraat

Norway	Oslo	Berit Flato
Poland	Bydgoszcz	Katarzyna Kobusinska
Poland	Kraków	Zbigniew Zuber
Poland	Wroclaw	Bogna Dobrzyniecka
Poland	Warsaw	Lidia Rutkowska Sak
Russia	St. Petersburg	Vyacheslav G. Chasnyk
Russia	Moscow	Nasonov or Nikishina
Serbia	Nis	Jelena Vojinovic
Serbia	Belgrade	Susic Gordana
Slovakia	Piestany	Elena Koskova
Slovenia	Ljubljana	Tadej Avcin
Spain	Esplugues (Barcelona)	Jordi Anton Lopez
Spain	Valencia	Inmaculada Calvo Penades
Spain	Madrid	Maria Luz Gamir

Supplementary Table S2. All baseline variables tested for association with CID – univariate analysis

		JADAS criteria			JIA-ACR criteria		
**Baseline variable**	**Category**	**n/responders (%)**	**OR [95% CI]**	***P* value**	**n/responders (%)**	**OR [95% CI]**	***P* value**
Sex female *vs* male	Female	53/72 (73.6)	0.95 (0.43–2.13)	0.9055	47/72 (65.3)	1.07 (0.52–2.24)	0.8480
	Male	41/55 (74.5)			35/55 (63.6)		
**Baseline weight Z score >1.24 *vs* ≤1.24**	**≤1.24**	76/107 (71.0)	3.67 (0.80–16.76)	0.0935	**64/107 (59.8)**	**6.05 (1.33–27.40)**	**0.0196**
	**>1.24**	18/20 (90.0)			**18/20 (90.0)**		
**Baseline BMI Z score >0.80 *vs* ≤0.80**	**≤0.80**	68/95 (71.6)	1.59 (0.58–4.31)	0.3646	**56/95 (58.9)**	**2.79 (1.04–7.45)**	**0.0412**
	**>0.80**	24/30 (80.0)			**24/30 (80.0)**		
Baseline height (Z score) >-0.74 *vs* ≤-0.74	≤-0.74	13/22 (59.1)	2.28 (0.87–5.98)	0.0943	11/22 (50.0)	2.03 (0.80–5.15)	0.1363
	>-0.74	79/103 (76.7)			69/103 (67.0)		
Disease duration (months) >28.62 *vs* ≤28.62	≤28.62	58/81 (71.6)	1.43 (0.61–3.34)	0.4123	48/81 (59.3)	1.95 (0.88–4.31)	0.0997
	>28.62	36/46 (78.3)			34/46 (73.9)		
**HLA-B27 positive *vs* negative**	**Positive**	**33/37 (89.2)**	**3.99 (1.29–12.32)**	**0.0163**	27/37 (73.0)	1.75 (0.75–4.06)	0.1923
	**Negative**	**60/89 (67.4)**			54/89 (60.7)		
Prorated number of active joints >11 *vs* ≤11	≤11	84/112 (75.0)	0.67 (0.21–2.13)	0.4915	71/112 (63.4)	1.59 (0.47–5.31)	0.4529
	>11	10/15 (66.7)			11/15 (73.3)		
Prorated number of joints with LOM >12 *vs* ≤12	≤12	83/116 (71.6)	300777.6 (0.00–5.04E228)	0.9616	71/116 (61.2)	1054823 (0.00,I)	0.9716
	>12	11/11 (100)			11/11 (100)		
Prorated number of painful joints >13 *vs* ≤13	≤13	87/117 (74.4)	0.81 (0.20–3.33)	0.7632	75/117 (64.1)	1.31 (0.32–5.32)	0.7089
	>13	7/10 (70.0)			7/10 (70.0)		
**Prorated number of swollen joints ≤4 *vs* >4**	**≤4**	**56/69 (81.2)**	**2.27 (1.05–5.10)**	**0.0478**	48/69 (69.6)	1.61 (0.78–3.36)	0.2003
	**>4**	**38/58 (65.5)**			34/58 (58.6)		
CRP (mg/L) ≤56.30 *vs* >56.30	≤56.30	92/123 (74.8)	2.97 (0.40–21.97)	0.2868	81/123 (65.9)	5.78 (0.58–57.32)	0.1336
	>56.30	2/4 (50.0)			1/4 (25.0)		
CHAQ score ≤1.13 *vs* >1.13	≤1.13	70/92 (76.1)	1.46 (0.62–3.44)	0.3895	63/92 (68.5)	1.83 (0.82–4.06)	0.1376
	>1.13	24/35 (68.6)			19/35 (54.3)		
Race White *vs* non-White	Non-White	8/12 (66.7)	1.48 (0.42–5.29)	0.5437	6/12 (50.0)	1.95 (0.59–6.44)	0.2741
	White	86/115 (74.8)			76/115 (66.1)		

		JADAS criteria			JIA-ACR criteria		
**Age at onset (years) ≤11.99 *vs* >11.99**	**≤11.99**	**67/82 (81.7)**	**2.98 (1.31–6.75)**	**0.0089**	**61/82 (74.4)**	**3.32 (1.54–7.15)**	**0.0022**
	**>11.99**	**27/45 (60.0)**			**21/45 (46.7)**		
Morning stiffness (min) >170 *vs* ≤170	≤170	82/112 (73.2)	1.46 (0.39–5.55)	0.5756	71/112 (63.4)	1.59 (0.47–5.31)	0.4529
	>170	12/15 (80.0)			11/15 (73.3)		
Tender entheseal score ≤1.0 *vs* >1.0	≤1.0	10/14 (71.4)	0.66 (0.14–3.03)	0.5896	9/14 (64.3)	1.08 (0.27–4.25)	0.9124
	>1.0	19/24 (79.2)			15/24 (62.5)		
Overall back pain VAS (mm) ≤44 *vs* >44	≤44	23/28 (82.1)	1.53 (0.24–9.95)	0.6539	20/28 (71.4)	2.50 (0.50–12.51)	0.2648
	>44	6/8 (75.0)			4/8 (50.0)		
Nocturnal back pain VAS (mm) ≤35 *vs* >35	≤35	25/32 (78.1)	1.79 (0.27–11.86)	0.5483	22/32 (68.8)	4.40 (0.69–28.11)	0.1174
	>35	4/6 (66.7)			2/6 (33.3)		
Modified Schober's test (cm) ≤15.4 *vs* >15.4	≤15.4	19/26 (73.1)	0.60 (0.10–3.45)	0.5735	18/26 (69.2)	2.70 (0.63–11.51)	0.1794
	>15.4	9/11 (81.8)			5/11 (45.5)		
Psoriasis BSA (%) >10 *vs* ≤10	≤10	13/21 (61.9)	4.31 (0.44–41.80)	0.2079	12/21 (57.1)	2.25 (0.37–13.87)	0.3822
	>10	7/8 (87.5)			6/8 (75.0)		
PGA of psoriasis ≤2 *vs* >2	≤2	13/18 (72.2)	1.49 (0.30–7.39)	0.6286	13/18 (72.2)	3.12 (0.65–15.03)	0.1562
	>2	7/11 (63.6)			5/11 (45.5)		
Baseline DMARDs yes *vs* no	No	11/18 (61.1)	2.03 (0.71–5.78)	0.1837	11/18 (61.1)	1.19 (0.43–3.32)	0.7409
	Yes	83/109 (76.1)			71/109 (65.1)		
Baseline MTX yes *vs* no	No	27/41 (65.9)	1.83 (0.80–4.16)	0.1502	26/41 (63.4)	1.08 (0.50–2.34)	0.8513
	Yes	67/86 (77.9)			56/86 (65.1)		
Baseline sulfasalazine yes *vs* no	No	79/108 (73.1)	1.38 (0.42–4.49)	0.5963	68/108 (63.0)	1.65 (0.55–4.91)	0.3712
	Yes	15/19 (78.9)			14/19 (73.7)		
Baseline chloroquine yes *vs* no	No	94/126 (74.6)	0.00 (0.00–I)	0.9885	82/126 (65.1)	0.00 (0.00–1)	0.9861
	Yes	0/1 (0)			0/1 (0)		
Baseline hydroxychloroquine no *vs* yes	No	93/124 (75.0)	6.00 (0.53–68.45)	0.1492	81/124 (65.3)	3.76 (0.33–42.69)	0.2847
	Yes	1/3 (33.3)			1/3 (33.3)		
Baseline oral corticosteroids yes *vs* no	No	81/111 (73.0)	1.60 (0.43–6.03)	0.4835	71/111 (64.0)	1.24 (0.40–3.82)	0.7087
	Yes	13/16 (81.3)			11/16 (68.8)		
Baseline oral NSAIDs yes *vs* no	No	44/59 (74.6)	0.94 (0.43–2.08)	0.8933	38/59 (64.4)	1.01 (0.49–2.10)	0.9720
	Yes	50/68 (73.5)			44/68 (64.7)		

		JADAS criteria			JIA-ACR criteria		
CRP normal *vs* high	High	23/32 (71.9)	1.16 (0.47–2.84)	0.7496	22/32 (68.8)	0.78 (0.33–1.82)	0.5678
	Normal	71/95 (74.7)			60/95 (63.2)		
Uveitis (from AE data set) yes *vs* no	No	80/112 (71.4)	285711.1 (0.00–I)	0.9832	71/112 (63.4)	350539.3 (0.00–I)	0.9815
	Yes	2/2 (100)			2/2 (100)		
**Patient/Parent Global Assessment ≤2.5 *vs* >2.5**	**≤2.5**	24/28 (85.7)	2.49 (0.79–7.80)	0.1186	**23/28 (82.1)**	**3.12 (1.09–8.89)**	**0.0333**
	**>2.5**	70/99 (70.7)			**59/99 (59.6)**		
CHAQ ≤1 *vs* >1 (1014)	≤1	65/85 (76.5)	1.46 (0.64–3.32)	0.3708	58/85 (68.2)	1.61 (0.75–3.45)	0.2205
	>1	29/42 (69.0)			24/42 (57.1)		
CHAQ >0.75 *vs* ≤0.75 (1014)	≤0.75	49/67 (73.1)	1.10 (0.50–2.44)	0.8109	43/67 (64.2)	1.04 (0.50–2.15)	0.9231
	>0.75	45/60 (75.0)			39/60 (65.0)		
CHAQ ≤1 *vs* >1 (1023)	≤1	65/85 (76.5)	1.46 (0.64–3.32)	0.3708	58/85 (68.2)	1.61 (0.75–3.45)	0.2205
	>1	29/42 (69.0)			24/42 (57.1)		
CHAQ >0.75 *vs* ≤0.75 (1023)	≤0.75	49/67 (73.1)	1.10 (0.50–2.44)	0.8109	43/67 (64.2)	1.04 (0.50–2.15)	0.9231
	>0.75	45/60 (75.0)			39/60 (65.0)		
Number of joints with active arthritis >11 *vs* ≤11	≤11	84/112 (75.0)	0.67 (0.21–2.13)	0.4915	71/112 (63.4)	1.59 (0.47–5.31)	0.4529
	>11	10/15 (66.7)			11/15 (73.3)		
Number of joints with limited range of motion 0 *vs* 1	0	85/113 (75.2)	1.69 (0.52–5.46)	0.3825	76/113 (67.3)	2.74 (0.89–8.47)	0.0804
	1	9/14 (64.3)			6/14 (42.9)		
**JADAS CID (≤1) at 3 months yes *vs* no**	**No**	74/102 (72.5)	311262.1 (0.00–2.9E187)	0.9528	**64/102 (62.7)**	**10.09 (1.29–78.87)**	**0.0276**
	**Yes**	18/18 (100)			**17/18 (94.4)**		
JADAS CID (≤1) at Week 8 yes *vs* no	No	85/114 (74.6)	274764.3 (0.00–1.44E294)	0.9705	73/114 (64.0)	960110.7 (0.00–I)	0.9778
	Yes	7/7 (100)			7/7 (100)		
JADAS CID (≤1) at Week 4 yes *vs* no	No	90/121 (74.4)	270079.1 (0.00–I)	0.9775	78/121 (64.5)	933738.4 (0.00–I)	0.9831
	Yes	4/4 (100)			4/4 (100)		
**JADAS LDA at 3 months yes *vs* no**	**No**	**49/74 (66.2)**	**7.31 (2.06–25.93)**	**0.0021**	**41/74 (55.4)**	**5.36 (2.03–14.19)**	**0.0007**
	**Yes**	**43/46 (93.5)**			**40/46 (87.0)**		
JIA-ACR (Wallace) CID at 3 months yes *vs* no	No	79/108 (73.1)	299489.2 (0.00–9.8E203)	0.9569	67/108 (62.0)	1056827 (0.00–7.06E294)	0.9674
	Yes	15/15 (100)			15/15 (100)		
JIA-ACR (Wallace) CID at Week 8 yes *vs* no	No	89/118 (75.4)	262415.3 (0.00–I)	0.9808	77/118 (65.3)	334856.1 (0.00–I)	0.9778
	Yes	3/3 (100)			3/3 (100)		

		JADAS criteria			JIA-ACR criteria		
JIA-ACR (Wallace) CID at Week 4 yes *vs* no	No	91/123 (74.0)	270709.6 (0.00–I)	0.9803	79/123 (64.2)	934903.6 (0.00–I)	0.9853
	Yes	3/3 (100)			3/3 (100)		
CRP at 3 months normal *vs* high	High	2/4 (50.0)	3.46 (0.46–25.78)	0.2255	3/4 (75.0)	1.46 (0.15–14.52)	0.7461
	Normal	90/116 (77.6)			78/116 (67.2)		

Bold font indicates significant factors shown in Figure 1.

AE: adverse event; BSA: body surface area; CID: clinically inactive disease; CHAQ: Childhood Health Assessment Questionnaire; CR: clinical remission; I: infinite; JADAS: Juvenile Arthritis Disease Activity Score; LDA: low disease activity; LOM: limitation of motion; OR: odds ratio; PGA: Physician's Global Assessment; VAS, visual analogue scale.

Supplementary Table S3. All baseline variables tested for association with CR – univariate analysis

		JADAS criteria			JIA-ACR criteria		
**Baseline variable**	**Category**	**n/responders (%)**	**OR [95% CI]**	***P* value**	**n/responders (%)**	**OR [95% CI]**	***P* value**
Sex female *vs* male	Female	26/72 (36.1)	1.38 (0.65–2.93)	0.4054	18/72 (25.0)	1.19 (0.52–2.75)	0.6759
	Male	16/55 (29.1)			12/55 (21.8)		
Baseline weight Z score ≤0.09 *vs* >-0.09	≤0.09	18/49 (36.7)	1.31 (0.61–2.78)	0.4871	16/49 (32.7)	2.22 (0.97–5.09)	0.0606
	>-0.09	24/78 (30.8)			14/78 (17.9)		
**Baseline BMI Z score ≤-0.28 *vs* >-0.28**	**≤-0.28**	19/45 (42.2)	1.93 (0.89–4.16)	0.0945	**15/45 (33.3)**	**2.36 (1.01–5.50)**	**0.0471**
	**>-0.28**	22/80 (27.5)			**14/80 (17.5)**		
Baseline height (Z score) >1.23 *vs* ≤1.23	≤1.23	33/106 (31.1)	1.61 (0.59–4.37)	0.3509	22/106 (20.8)	2.23 (0.78–6.32)	0.1326
	>1.23	8/19 (42.1)			7/19 (36.8)		
**Disease duration (months) ≤6.83 *vs* >6.83**	**≤6.83**	12/27 (44.4)	1.87 (0.78–4.46)	0.1603	**11/27 (40.7)**	**2.93 (1.18–7.14)**	**0.0214**
	**>6.83**	30/100 (30.0)			**19/100 (19.0)**		
**HLA-B27 positive *vs* negative**	**Positive**	**19/37 (51.4)**	**3.03 (1.36–6.74)**	**0.0067**	**15/37 (40.5)**	**3.36 (1.42–7.94)**	**0.0057**
	**Negative**	**23/89 (25.8)**			**15/89 (16.9)**		
Prorated number of active joints >6 *vs* ≤6	≤6	24/75 (32.0)	1.13 (0.53–2.38)	0.7581	16/75 (21.3)	1.36 (0.60–3.10)	0.4666
	>6	18/52 (34.6)			14/52 (26.9)		
Prorated number of joints with LOM >5 *vs* ≤5	≤5	25/74 (33.8)	0.93 (0.44–1.96)	0.8401	16/74 (21.6)	1.30 (0.57–2.97)	0.5311
	>5	17/53 (32.1)			14/53 (26.4)		
Prorated number of painful joints ≤2 *vs* >2	≤2	12/25 (48.0)	2.22 (0.91–5.41)	0.0808	8/25 (32.0)	1.71 (0.65–4.49)	0.2743
	>2	30/102 (29.4)			22/102 (21.6)		
Prorated number of swollen joints >12 *vs* ≤12	≤12	38/119 (31.9)	2.13 (0.51–8.98)	0.3025	27/119 (22.7)	2.04 (0.46–9.11)	0.3482
	>12	4/8 (50.0)			3/8 (37.5)		
CRP (mg/L) ≤8.7 *vs* >8.7	≤8.7	34/97 (35.1)	1.48 (0.60–3.69)	0.3954	25/97 (25.8)	1.74 (0.60–5.02)	0.3089
	>8.7	8/30 (26.7)			5/30 (16.7)		
CHAQ score >0.87 *vs* ≤0.87	≤0.87	22/67 (32.8)	1.02 (0.49–2.14)	0.9525	12/67 (17.9)	1.96 (0.85–4.52)	0.1124
	>0.87	20/60 (33.3)			18/60 (30.0)		
Race White *vs* non-White	Non-White	3/12 (25.0)	1.54 (0.39–6.01)	0.5350	3/12 (25.0)	0.92 (0.23–3.70)	0.9060
	White	39/115 (33.9)			27/115 (23.5)		

		JADAS criteria			JIA-ACR criteria		
**Age at onset (years) ≤2.94 *vs* >2.94**	**≤2.94**	11/23 (47.8)	2.16 (0.86–5.42)	0.1010	**11/23 (47.8)**	**4.10 (1.57–10.68)**	**0.0039**
	**>2.94**	31/104 (29.8)			**19/104 (18.3)**		
**Morning stiffness (min) >250 *vs* ≤250**	**≤250**	**37/120 (30.8)**	**5.61 (1.04–30.24)**	**0.0449**	**26/120 (21.7)**	**4.82 (1.01–22.91)**	**0.0480**
	**>250**	**5/7 (71.4)**			**4/7 (57.1)**		
Tender entheseal score ≤10 *vs* >10	≤10	12/33 (36.4)	138312.2 (0.00–2.66E192)	0.9571	8/33 (24.2)	105266.6 (0.00–2.27E223)	0.9640
	>10	0/5 (0)			0/5 (0.0)		
Overall back pain VAS (mm) >9 *vs* ≤9	≤9	6/17 (35.3)	0.99 (0.25–3.82)	0.9851	4/17 (23.5)	1.08 (0.24–4.90)	0.9173
	>9	7/20 (35.0)			5/20 (25.0)		
Nocturnal back pain VAS (mm) >35 *vs* ≤35	≤35	10/32 (31.3)	1.10 (0.17–7.03)	0.9198	6/32 (18.8)	2.17 (0.32–14.71)	0.4289
	>35	2/6 (33.3)			2/6 (33.3)		
Modified Schober's test (cm) ≤14.2 *vs* >14.2	≤14.2	3/10 (30.0)	1.02 (0.21–4.97)	0.9825	3/10 (30.0)	1.89 (0.36–9.97)	0.4553
	>14.2	8/27 (29.6)			5/27 (18.5)		
Psoriasis BSA (%) >4 *vs* ≤4	≤4	4/14 (28.6)	0.91 (0.18–4.64)	0.9087	1/14 (7.1)	2.00 (0.16–24.87)	0.5899
	>4	4/15 (26.7)			2/15 (13.3)		
PGA of psoriasis >2 *vs* ≤2	≤2	4/18 (22.2)	2.00 (0.38–10.48)	0.4121	1/18 (5.6)	3.78 (0.30–47.55)	0.3037
	>2	4/11 (36.4)			2/11 (18.2)		
Baseline DMARDs yes *vs* no	No	0/18 (0)	1088022 (0.00–2.27E270)	0.9643	0/18 (0.0)	1007524 (0.00–3.9E175)	0.9526
	Yes	42/109 (38.5)			30/109 (27.5)		
**Baseline MTX yes *vs* no**	**No**	**8/41 (19.5)**	**2.70 (1.11–6.54)**	**0.0280**	6/41 (14.6)	2.26 (0.84–6.05)	0.1054
	**Yes**	**34/86 (39.5)**			24/86 (27.9)		
Baseline sulfasalazine yes *vs* no	No	34/108 (31.5)	1.58 (0.58–4.29)	0.3667	24/108 (22.2)	1.62 (0.56–4.70)	0.3787
	Yes	8/19 (42.1)			6/19 (31.6)		
Baseline chloroquine yes *vs* no	No	42/126 (33.3)	0.00 (0.00–I)	0.9873	30/126 (23.8)	0.00 (0.00–I)	0.9890
	Yes	0/1 (0.0)			0/1 (0.0)		
Baseline hydroxychloroquine yes *vs* no	No	42/124 (33.9)	0.00 (0.00–I)	0.9856	30/124 (24.2)	0.00 (0.00–I)	0.9809
	Yes	0/3 (0.0)			0/3 (0.0)		
Baseline oral corticosteroids yes *vs* no	No	36/111 (32.4)	1.25 (0.42–3.71)	0.6875	26/111 (23.4)	1.09 (0.32–3.67)	0.8896
	Yes	6/16 (37.5)			4/16 (25.0)		
Baseline oral NSAIDs no *vs* yes	No	24/59 (40.7)	1.90 (0.90–4.03)	0.0915	16/59 (27.1)	1.44 (0.63–3.26)	0.3887
	Yes	18/68 (26.5)			14/68 (20.6)		

		JADAS criteria			JIA-ACR criteria		
CRP normal *vs* high	High	9/32 (28.1)	1.36 (0.56–3.28)	0.4926	6/32 (18.8)	1.46 (0.54–3.99)	0.4550
	Normal	33/95 (34.7)			24/95 (25.3)		
Uveitis (from AE data set) yes *vs* no	No	34/112 (30.4)	1728941 (0.00–I)	0.9813	24/112 (21.4)	5555237 (0.00–I)	0.9858
	Yes	2/2 (100.0)			2/2 (100.0)		
**Patient/Parent Global Assessment ≤2.5 *vs* >2.5**	**≤2.5**	**15/28 (53.6)**	**3.08 (1.30–7.30)**	**0.0108**	**11/28 (39.3)**	**2.72 (1.10–6.76)**	**0.0306**
	**>2.5**	**27/99 (27.3)**			**19/99 (19.2)**		
CHAQ ≤1 *vs* >1 (1014)	≤1	32/85 (37.6)	1.93 (0.84–4.45)	0.1220	20/85 (23.5)	0.98 (0.41–2.35)	0.9721
	>1	10/42 (23.8)			10/42 (23.8)		
CHAQ >0.75 *vs* ≤0.75 (1014)	≤0.75	22/67 (32.8)	1.02 (0.49–2.14)	0.9525	12/67 (17.9)	1.96 (0.85–4.52)	0.1124
	>0.75	20/60 (33.3)			18/60 (30.0)		
CHAQ ≤1 *vs* >1 (1023)	≤1	32/85 (37.6)	1.93 (0.84–4.45)	0.1220	20/85 (23.5)	0.98 (0.41–2.35)	0.9721
	>1	10/42 (23.8)			10/42 (23.8)		
CHAQ >0.75 *vs* ≤0.75 (1023)	≤0.75	22/67 (32.8)	1.02 (0.49–2.14)	0.9525	12/67 (17.9)	1.96 (0.85–4.52)	0.1124
	>0.75	20/60 (33.3)			18/60 (30.0)		
Number of joints with active arthritis >6 *vs* ≤6	≤6	24/75 (32.0)	1.13 (0.53–2.38)	0.7581	16/75 (21.3)	1.36 (0.60–3.10)	0.4666
	>6	18/52 (34.6)			14/52 (26.9)		
Number of joints with limited range of motion 0 *vs* 1	0	39/113 (34.5)	1.93 (0.51–7.34)	0.3332	27/113 (23.9)	1.15 (0.30–4.43)	0.8378
	1	3/14 (21.4)			3/14 (21.4)		
**JADAS CID (≤1) at 3 months yes *vs* no**	**No**	**29/102 (28.4)**	**5.03 (1.73–14.68)**	**0.0031**	**19/102 (18.6)**	**5.46 (1.90–15.68)**	**0.0016**
	**Yes**	**12/18 (66.7)**			**10/18 (55.6)**		
**JADAS CID (≤1) at Week 8 yes *vs* no**	**No**	**34/114 (29.8)**	**14.12 (1.64–121.77)**	**0.0160**	**24/114 (21.1)**	**5.00 (1.05–23.87)**	**0.0436**
	**Yes**	**6/7 (85.7)**			**4/7 (57.1)**		
JADAS CID (≤1) at Week 4 yes *vs* no	No	39/121 (32.2)	6.31 (0.64–62.60)	0.1158	28/121 (23.1)	3.32 (0.45–24.66)	0.2406
	Yes	3/4 (75.0)			2/4 (50.0)		
**JADAS LDA at 3 months yes *vs* no**	**No**	**16/74 (21.6)**	**4.32 (1.94–9.62)**	**0.0004**	**9/74 (12.2)**	**5.56 (2.24–13.78)**	**0.0002**
	**Yes**	**25/46 (54.3)**			**20/46 (43.5)**		
**JIA-ACR (Wallace) CID at 3 months yes *vs* no**	**No**	**32/108 (29.6)**	**4.75 (1.50–15.00)**	**0.0079**	**22/108 (20.4)**	**4.47 (1.46–13.65)**	**0.0086**
	**Yes**	**10/15 (66.7)**			**8/15 (53.3)**		
JIA-ACR (Wallace) CID at Week 8 yes *vs* no	No	37/118 (31.4)	1547961 (0.00–I)	0.9766	25/118 (21.2)	5407359 (0.00–I)	0.9822
	Yes	3/3 (100.0)			3/3 (100.0)		

		JADAS criteria			JIA-ACR criteria		
JIA-ACR (Wallace) CID at Week 4 yes *vs* no	No	39/123 (31.7)	1506674 (0.00–I)	0.9765	27/123 (22.0)	4779159 (0.00–I)	0.9817
	Yes	3/3 (100.0)			3/3 (100.0)		
CRP at 3 months normal *vs* high	High	1/4 (25.0)	1.58 (0.16–15.67)	0.6967	0/4 (0.0)	266701.0 (0.00–I)	0.9777
	Normal	40/116 (34.5)			29/116 (25.0)		

Bold font indicates significant factors shown in Figure 3.

AE: adverse event; BSA: body surface area; CID: clinically inactive disease; CHAQ: Childhood Health Assessment Questionnaire; CR: clinical remission; I: infinite; JADAS: Juvenile Arthritis Disease Activity Score; LDA: low disease activity; LOM: limitation of motion; OR: odds ratio; PGA: Physician's Global Assessment; VAS, visual analogue scale.

Supplementary Table S4. Significant predictors for CID – multivariate analysis

Predictor	JADAS criteria		ACR Wallace criteria	
	**OR [95% CI]**	***P* value**	**OR [95% CI]**	***P* value**
Baseline height (Z score) >-0.74 *vs* ≤-0.74	6.526 [1.554–27.399]	0.010	-	-
Baseline BMI (Z score) >0.80 *vs* ≤0.80	-	-	5.085 [1.444–17.902]	0.011
Age at onset (years) ≤11.99 *vs* >11.99	13.154 [3.144–55.031]	<0.001	2.745 [1.064–7.077]	0.037
CRP normal *vs* high at 3 months	16.904 [1.122–254.743]	0.041	3.699 [1.165–11.750]	0.027
HLA-B27 positive *vs* negative	7.942 [1.661–37.985]	0.010	-	-
JADAS LDA at 3 months yes *vs* no	11.636 [2.143–63.192]	0.005	7.154 [2.312–22.131]	<0.001
Prorated number of swollen joints ≤4 *vs* >4	4.260 [1.277–14.213]	0.018	-	-

CID: clinically inactive disease; CR: clinical remission; JADAS: Juvenile Arthritis Disease Activity Score; LDA, low disease activity.

Supplementary Table S5. Significant predictors for CR – multivariate analysis

Predictor	JADAS criteria		ACR Wallace criteria	
	**OR [95% CI]**	***P* value**	**OR [95% CI]**	***P* value**
Baseline height (Z score) >1.23 *vs* ≤1.23	-	-	17.365 [3.103–97.167]	0.001
JADAS LDA at 3 months yes *vs* no	4.354 [1.891–10.025]	<0.001	18.563 [4.010–85.937]	<0.001
Prorated number of swollen joints >12.00 *vs* ≤12.00	-	-	20.976 [2.334–188.48]	0.007

CID: clinically inactive disease; CR: clinical remission; JADAS: Juvenile Arthritis Disease Activity Score; LDA, low disease activity.

Supplementary Figure S1. Subject disposition CLIPPER 2 (For CLIPPER see Horneff G, et al. Ann Rheum Dis 2014;73:1114–1122)

109 participants enrolled in CLIPPER 2 extension study

- 99 in Active Treatment Period + 10 in Observational Period from (Study B1801014)
- 55 eoJIA, 31 ERA, and 23 PsA

25 participants discontinued permanently

- 14 no longer willing to participate
- 4 lost to follow-up
- 2 for other reasons (at investigator's discretion)
- 1 insufficient clinical response
- 1 adverse event
- 1 protocol violation
- 1 withdrawn due to pregnancy
- 1 medication error without associated adverse event

84 participants completed study

- 27 completed study in Active Treatment Period
- 45 completed study in Observational Period
- 5 completed study in Re-Treatment Period
- 7 completed study in Withdrawal Period

Active Treatment Period

99 participants enrolled

- 27 completed study
- 30 to Withdrawal Period
- 29 to Observational Period
- 13 permanently discontinued

Observational Period

43 participants enrolled + 10 participants from CLIPPER

- 45 completed study
- 8 permanently discontinued

Re-Treatment Period

13 participants enrolled

- 5 completed study
- 7 to Observational Period
- 1 permanently discontinued

Withdrawal Period

30 participants enrolled

- 7 completed study
- 13 to Re-Treatment Period
- 7 to Observational Period
- 3 permanently discontinued

Note: Total discontinued is the sum of individual reasons since they are mutually exclusive by participant.
